# A novel prognostic model of breast cancer based on cuproptosis-related lncRNAs

**DOI:** 10.1007/s12672-024-00888-3

**Published:** 2024-02-14

**Authors:** Feixiang Li, Yongyan Yang, Xuan Zhang, Jiafeng Yu, Yonghao Yu

**Affiliations:** 1 Department of Anesthesiology, Tianjin Medical University General Hospital, NO.154, Anshan Road, Heping District, Tianjin, 300052 China; 2 Tianjin Research Institute of Anesthesiology, Tianjin, China; 3 Department of Anesthesiology, Tianjin Medical University Cancer Institute & Hospital, Tianjin, China; 4 Key Laboratory of Breast Cancer Prevention and Therapy, Tianjin Medical University, Ministry of Education, Tianjin, China

**Keywords:** Cuproptosis, lncRNAs, Prognostic model, Breast cancer, Bioinformatics

## Abstract

**Objective:**

Breast cancer (BC) is a deadly form of malignancy responsible for the death of a large number of women every year. Cuproptosis is a newly discovered form of cell death that may have implications for the prognosis of BC. Long non‐coding RNAs (lncRNAs) have been shown to be involved in the progression and development of BC. Here within, a novel model capable of predicting the prognosis of patients with BC was established based on cuproptosis-related lncRNAs.

**Methods:**

Data of breast cancer patients was downloaded, including clinical information from The Cancer Genome Atlas (TCGA) database and lncRNAs related to cuproptosis were isolated. In total, nine lncRNAs related to copper death were obtained by Cox regression model based on Least Absolute Shrinkage and Selector Operation (LASSO) algorithm for model construction. The model was verified by overall survival (OS), progression-free survival (PFS) and receiver operating characteristic (ROC) curve. The differences in immune function, tumor mutation burden (TMB) and tumor immune dysfunction and exclusion (TIDE) between patients with different risk scores were analyzed.

**Results:**

Based on cuproptosis-related lncRNAs, a prognostic model for predicting BC was constructed. Each patient was assigned a risk score based on our model formula. We found that patients with higher risk scores had significantly lower OS and PFS, increased TMB, and higher sensitivity to immunotherapy.

**Conclusions:**

The model established in this study based on cuproptosis-related lncRNAs may be capable of improving the OS of patients with BC.

**Supplementary Information:**

The online version contains supplementary material available at 10.1007/s12672-024-00888-3.

## Introduction

Breast cancer (BC) is responsible for countless deaths every year and poses a substantial threat to women throughout the world [1, 2]. BC has become the highest incidence of cancer in the United States, and the incidence of BC in China is also gradually rising [3, 4]. Despite continuous improvements in diagnostic and treatment technology, the issue remains, and continued efforts to enhance our understanding of BC could prove critical [5, 6]. As clearly demonstrated, a more accurate model to assess the outcomes of BC is desperately needed.

There is a novel form of cell death called cuproptosis, that involves the destruction of specific mitochondrial metabolic enzymes, leading to a unique mechanism of cell death [7]. LncRNAs are non-coding RNAs sequences with more than 200 nucleotides that regulate gene expression by interacting with proteins and RNAs [8, 9]. Numerous studies have demonstrated that lncRNAs are associated with the progression of BC. The cuproptosis-related lncRNAs have been proven to be highly correlated with the occurrence and development of lung cancer or other tumors [10, 11]. However, the role that cuproptosis-related lncRNAs play in BC remains unclear [12–14].

With the continuous improvement of bioinformatics technology, more and more predictive models based on bioinformatics analysis are used to assess cancer outcomes [15, 16], including models associated with ferroptosis and glycolysis to evaluate the prognosis of BC [17–19]. Representing a novel approach to this problem, BC prognostic prediction models relating to cuproptosis have not been investigated. Therefore, we constructed a novel predictive model of clinical prognosis in BC using cuproptosis-associated lncRNAs.

## Material and methods

### Data sources

Transcriptome files and clinical information for patients with BC were downloaded from The Cancer Genome Atlas (TCGA) [20].

### Identification of cuproptosis-related lncRNAs

Throughout this study, NFE2L2, NLRP3, ATP7B, ATP7A, SLC31A1, FDX1, LIAS, LIPT1,LIPT2, DLD, DLAT, PDHA1, PDHB, MTF1, GLS, CDKN2A, DBT, GCSH and DLST were selected as cuproptosis-related genes for subsequent analysis [10, 11, 21]. Initially, "perl" software was utilized to divide the transcriptome data of patients into mRNAs and lncRNAs according to the downloaded annotation files. Following this, R software was used to extract transcriptome data of each patient, which was then merged against cuproptosis-related genes to obtain the expression levels of cuproptosis-related genes. Finally, the expression levels of the above obtained lncRNAs and cuproptosis-related genes were analyzed for co-expression, and cuproptosis-related lncRNAs were retained for subsequent analysis. Filter parameters were set as follows: correlation coefficient $=0.4$, correlation test $P< 0.001$.

### Construction of the cuproptosis-related lncRNAs-based prognostic model

The downloaded data were grouped randomly, with 50% of patient data used for model construction and 50% for model validation. First, univariate Cox regression analysis was used, optimized and cross-validated by the Least Absolute Shrinkage and Selector Operation (LASSO) algorithm through "glmnet" package, to identify cuproptosis-related lncRNAs associated with patient prognosis. Cuproptosis-related lncRNAs with P $<$ 0.05 were defined as candidate lncRNAs. Then, multivariate Cox regression analysis was conducted based on candidate prognostic lncRNAs to obtain the optimal prognostic model. The risk formula is Risk score $={\sum }_{i=1}^{n}coef*id$, where ‘coef’ is the coefficient, ‘id’ is the expression level of each selected lncRNA and ‘n’ is the number of lncRNAs involved in model construction. The risk score of each patient was obtained by the above formula, and the patients were divided into high and low risk groups according to the median value of the risk score.

### Survival and ROC analysis

Initially, Kaplan–Meier survival curve analysis was performed utilizing the "survival" and "survminer" packages to calculate the overall survival (OS). Then, we downloaded the progression free survival (PFS) data for all tumors from the TCGA database and calculated whether PFS differed between patients with different risk scores. Finally, we used the receiver operating characteristic (ROC) curve and C-index curve to evaluate the precision of the model in predicting patient’ outcomes. The area under the curve (AUC) was used to quantify the accuracy of the constructed model.

### Independent prognostic analysis

To determine whether the risk scores were independent of other risk factor, univariate and multivariate Cox regression analyses were utilized with the measurement standard set to P < 0.05.

### Principal component and nomogram analysis

We used the "limma" and "sactterplot3d" packages of R software to calculate principal component analysis (PCA) and evaluate the ability of model-related lncRNAs to identify patients with different risk scores, and then used the "rms" package to construct a nomogram to evaluate the potential clinical utility of this model. The nomogram has been scored based on risk score and clinical characteristics, and the total sum of the items was used to predict the OS of patients at 1, 3, and 5-year.

### Tumor mutation burden and survival analysis

We obtained the tumor mutation status of each sample through the data downloaded by TCGA, and calculated the difference of tumor mutation burden (TMB) between high and low risk groups with "limma" package. Patients were divided into two groups according to TMB, and survival analysis between the different mutation groups was conducted through the "survival" and "survminer" packages. Finally, survival analysis was performed in combination with the risk score of the patients.

### Difference in immune function

We first performed ssGSEA on transcriptome data using "gsva" package to obtain the ssGSEA score of different immune function sets of each patient, and then difference analysis using the "limma" package to obtain differences in immune function between high and low risk groups. We obtained the tumor immune dysfunction and exclusion (TIDE) score of each sample through http://tide.dfci.harvard.edu/, and then compared the difference in TIDE scores between the high and low risk groups by the "limma" package. The measurement criterion was P < 0.05.

### Relationship between risk score and drug sensitivity

To further observe the difference in potential treatment mode of high and low risk groups. We used the "pRRophetic" package to observe the difference of half maximal inhibitory concentration (IC50) between high and low risk group after treatment with 30 common anticancer drugs and the measurement criterion was P < 0.05 [22, 23].

## Results

### The identification of cuproptosis-related lncRNAs

**Fig. 1:**
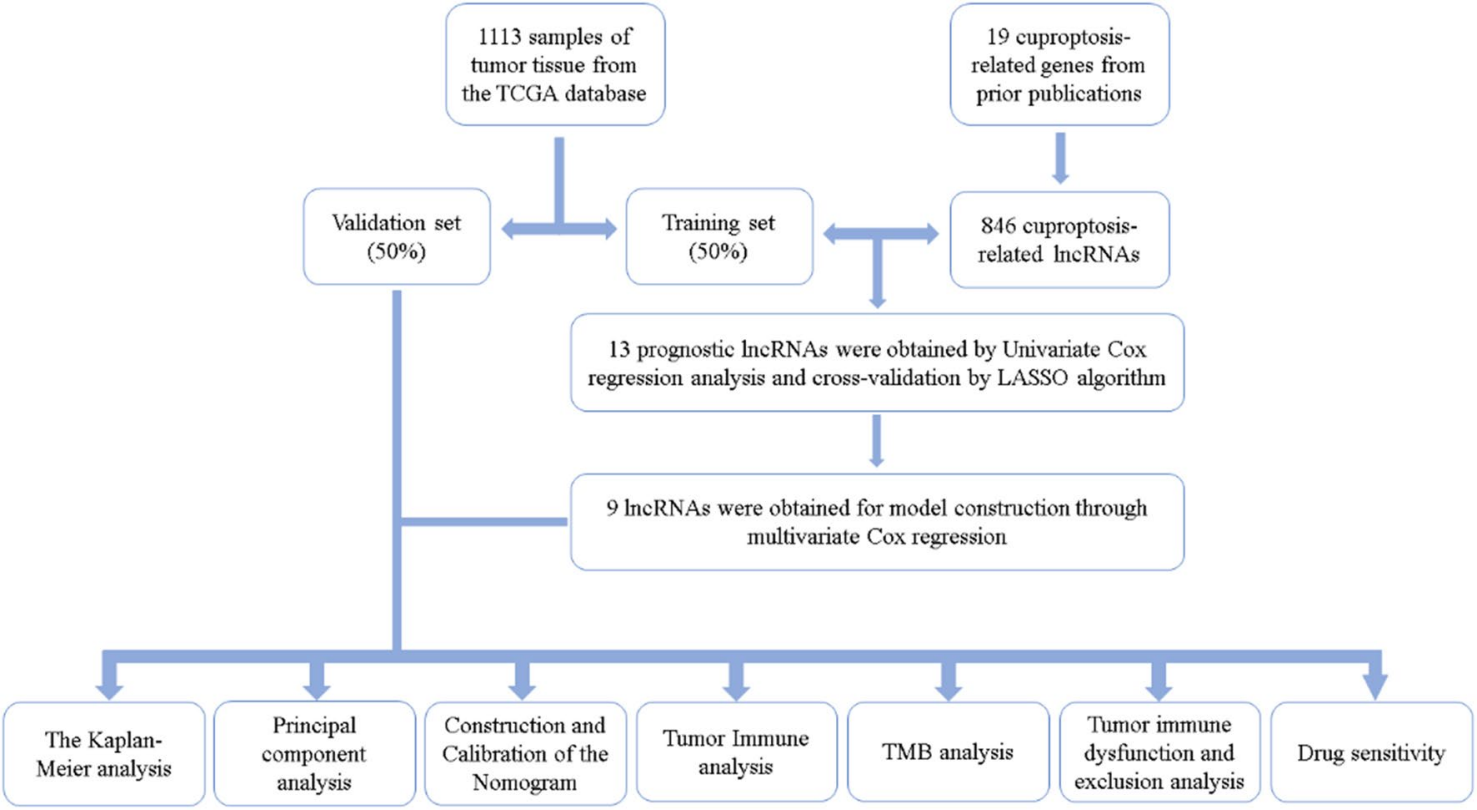
The overall flowchart of the experiment

**Fig. 2:**
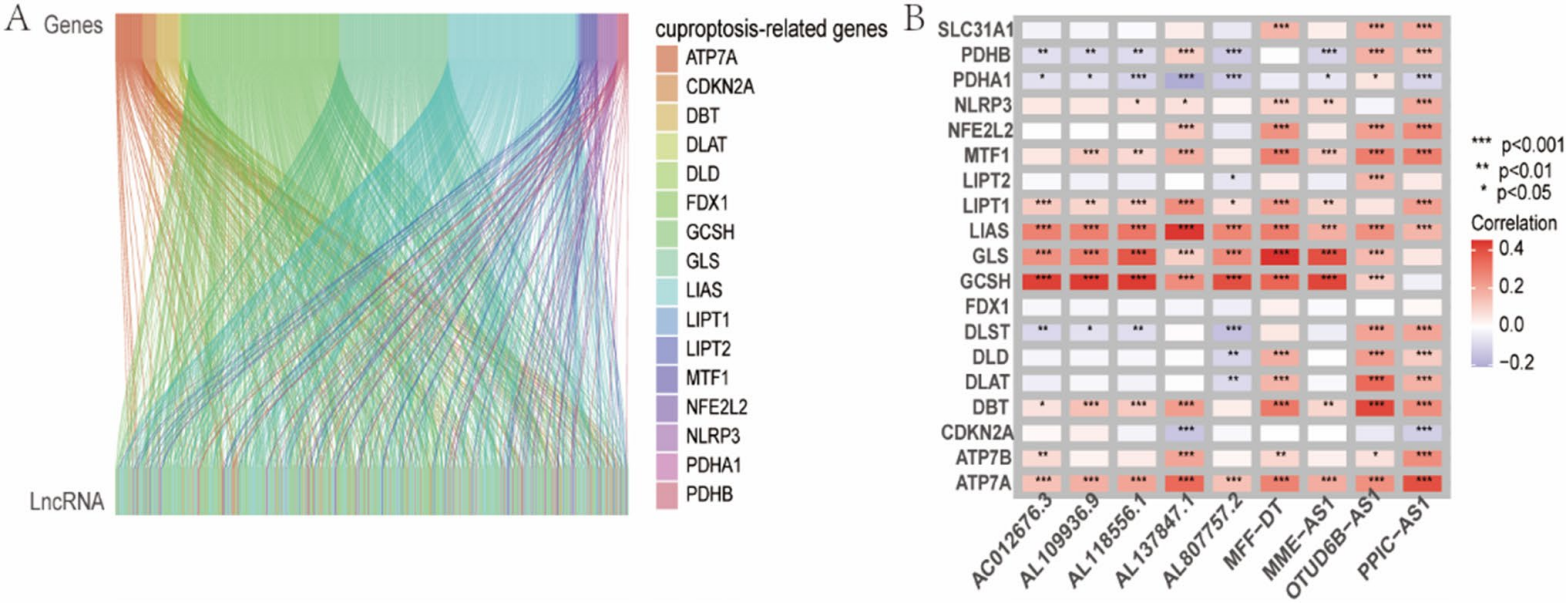
**A** The results of co-expression. Cuproptosis-related genes are shown on the right, cuproptosis-related genes are shown on the top, and lncRNAs are shown on the bottom. **B** The heatmap of the correlation between lncRNAs used for model construction and cuproptosis-related genes

Figure 1 shows the overall flowchart of the experiment. Firstly, 1113 samples of tumor tissue were downloaded from the TCGA database, all of which contained relevant clinical information. Through co-expression analysis of cuproptosis-related genes and lncRNAs, 846 lncRNAs related to cuproptosis were obtained, and their relationship with cuproptosis-related genes is shown in Fig. 2A.

### Prognostic model based on cuproptosis-related lncRNAs

Using the expression levels of the cuproptosis-related lncRNAs and the outcomes of patients, a prognostic model was constructed for BC. Firstly, 13 prognostic lncRNAs were obtained by univariate Cox regression analysis and cross-validation by LASSO algorithm (Table 1). The LASSO optimization results are shown in Additional file 1: Figure S1A, B. Following this, 9 lncRNAs were obtained for model construction through multivariate Cox regression (Table 1). The risk score for each patient was calculated by the formula: risk score = (1.14515 $*$ Expr PPIC-AS1) + (−1.94235 $*$ Expr MME-AS1) + (−0.54517 $*$ Expr AC012676.3) + (1.246856 $*$ Expr MFF-DT) + (0.83774 $*$ Expr OTUD6B-AS1) + (−0.49163 $*$ Expr AL109936.9) + (−0.87639 $*$ Expr AL807757.2) + (−1.34052 $*$ Expr AL118556.1) + (−2.04930 $*$ Expr AL137847.1). The heatmap of the correlation between lncRNAs used for model construction and cuproptosis-related genes is shown in Fig. 2B. The functional analysis results of the target lncRNA are shown in Additional file 1: Table S1.

**Table 1:** Results of univariate and multivariate cox regression analyses of cuproptosis-related lncRNAs

Univariate cox regression			Multivariate cox regression		
LncRNAs	HR	P	LncRNAs	HR	Coef
PPIC-AS1	1.869933	0.02831	PPIC-AS1	3.14292	1.145152
MME-AS1	0.058929	0.043736	MME-AS1	0.143366	−1.94235
AC012676.3	0.526084	0.018596	AC012676.3	0.579745	−0.54517
MFF-DT	5.759648	0.011792	MFF-DT	3.479392	1.246858
AP001021.1	0.145634	0.030604	OTUD6B-AS1	2.311141	0.837741
OTUD6B-AS1	1.531655	0.023922	AL109936.9	0.611631	−0.49163
AL109936.9	0.60987	0.028326	AL807757.2	0.416283	−0.87639
AC137932.1	0.631439	0.022652	AL118556.1	0.261709	−1.34052
AL807757.2	0.27515	0.010894	AL137847.1	0.128825	−2.0493
AP003696.1	0.113952	0.043774			
C8orf49	1.695612	0.018091			
AL118556.1	0.279904	0.011848			
AL137847.1	0.214887	0.005552			

### Model evaluation of nine cuproptosis-related lncRNAs

**Fig. 3:**
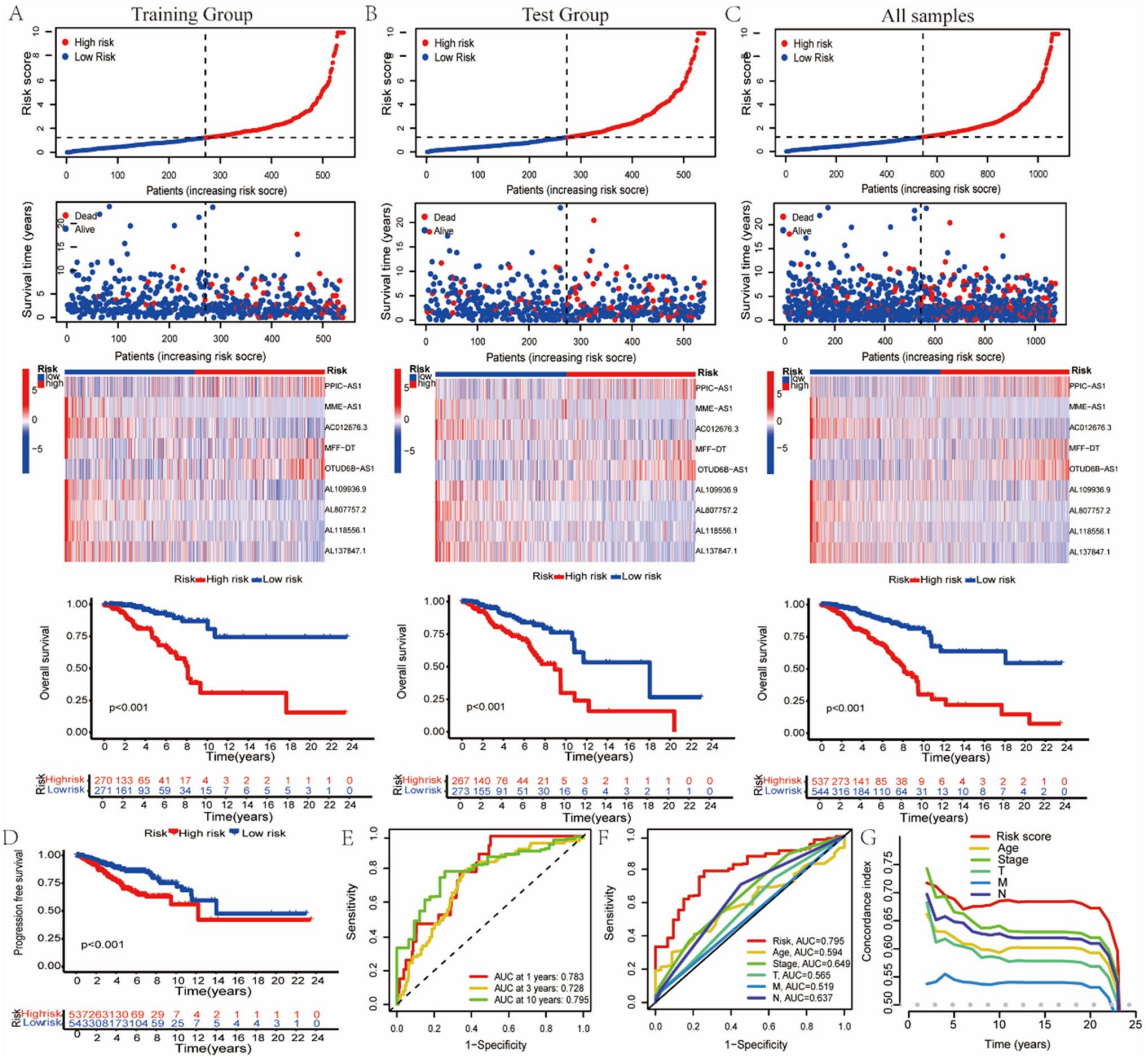
**A**, **B** and **C** show the distribution of patients' risk scores and survival status, heat maps of 9 cuproptosis -related lncRNAs expression and OS curves of the training group, test group and all samples, respectively. **D** PFS curves between different groups. **E** ROC curves for model prediction of 1, 3, and 10-year OS. (**F**) ROC curves of prognostic model and clinicopathological features. **G** Results of C-index curve

Patients were stratified based on their median risk scores into different groups through the prognostic model which was constructed based on nine cuproptosis-related lncRNAs. From the distribution of patients’ risk scores and survival status, we found that patients with higher risk scores died more frequently (Fig. 3A–C). We also found that high-risk patients had lower OS (Fig. 3A–C). The expression heatmap of nine cuproptosis-related lncRNAs is shown in Fig. 3A–C. We also compared PFS between the two groups and showed that patients with higher risk scores had lower PFS (Fig. 3D). To assess the precision of the model, we calculated the ROC value of the model to evaluate the patients’OS in 1, 3 and 10 years, and compared it with the clinical characteristics of patients, and found that our model was highly accurate (Fig. 3E, F). The results of C-index curve also suggest that our model was more precise in evaluating prognosis than clinicopathological features (Fig. 3G).

### Independent prognostic analysis and subgroup validation

**Fig. 4:**
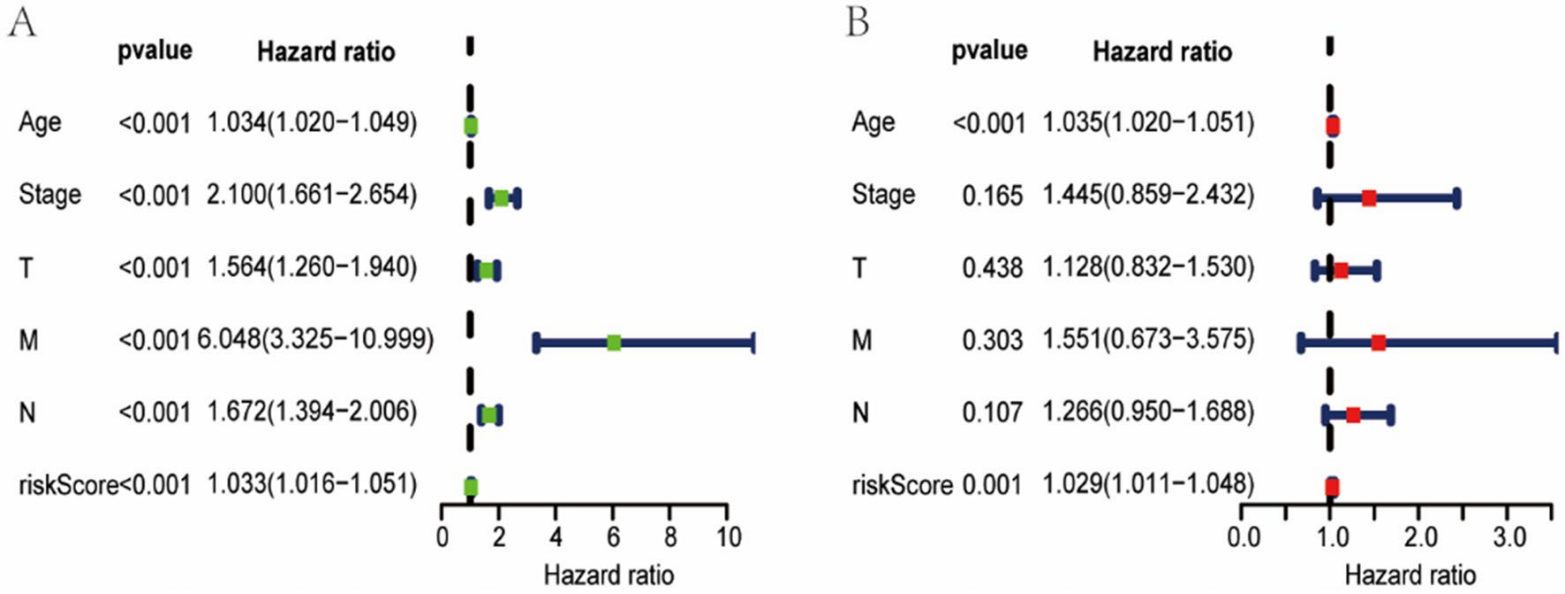
**A** and **B** show the univariate and multivariate Cox regression analyses

**Fig. 5:**
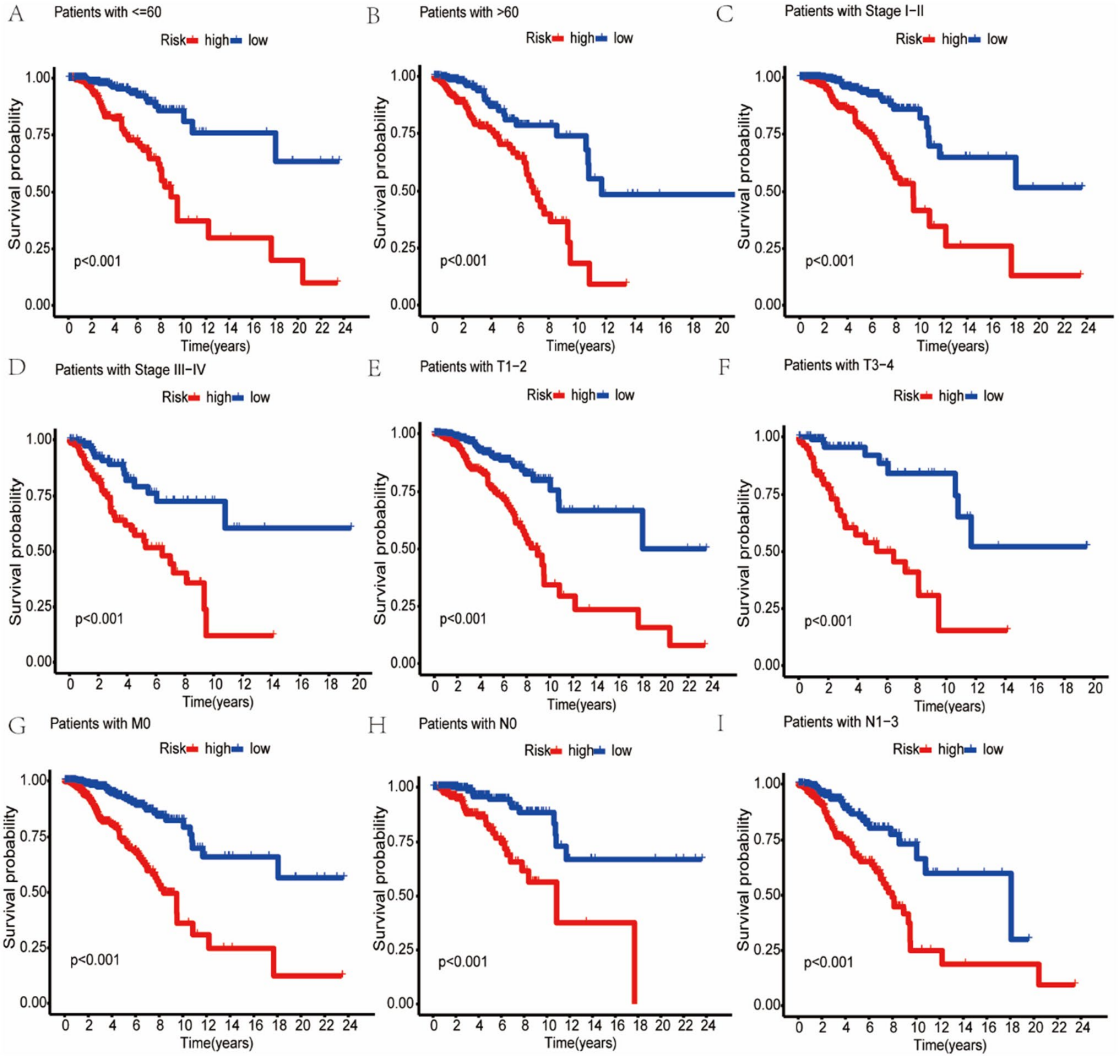
Survival analysis for BC in the subgroups of **A** age $\le$ 60, **B** age $>$ 60, **C** AJCC I-II stage, **D** AJCC III-IV stage, **E** T 1–2 stage, **F** T 3–4 stage, **G** M 0 stage, **H** N 0 stage, **I** N 1–3 stage

Cox regression analysis was performed to identify if the risk scores were independent of other risk factors in determining prognosis of BC. We found that risk score can be used to assess the prognosis of patients with BC independently, as shown in Fig. 4A, B. In addition, Kaplan–Meier survival analysis were also used for subgroups of patients, showing that patients with higher risk scores had significantly poorer outcomes in age, American Joint Committee on Cancer (AJCC), T, M, and N subgroups (Fig. 5A–I).

### Results of principal component and nomogram analyses

**Fig. 6:**
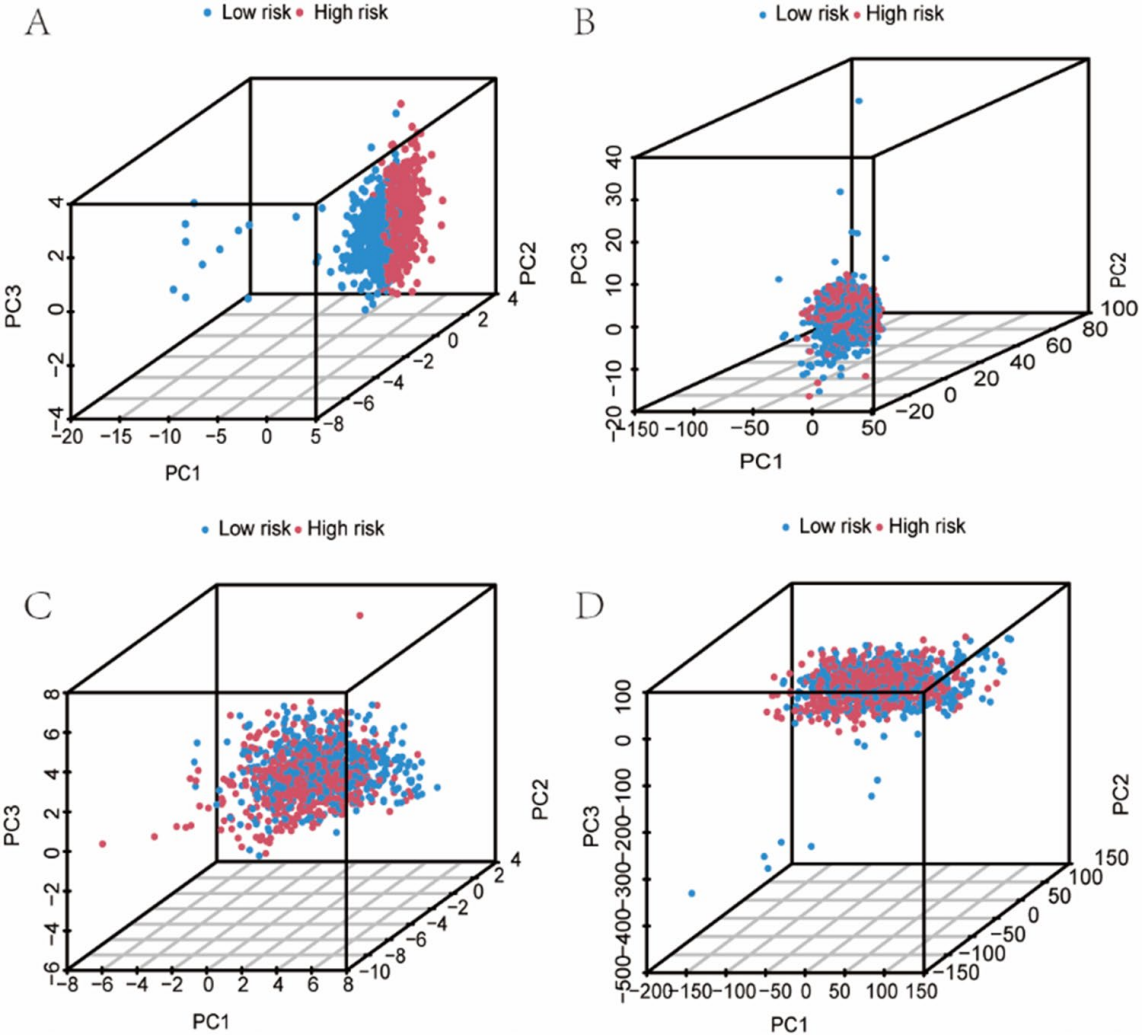
**A**, **B**, **C** and **D** respectively demonstrated the distinguishing ability of lncRNAs involved in model construction, cuproptosis-related lncRNAs, cuproptosis-related genes and all genes between different risk scores

**Fig. 7:**
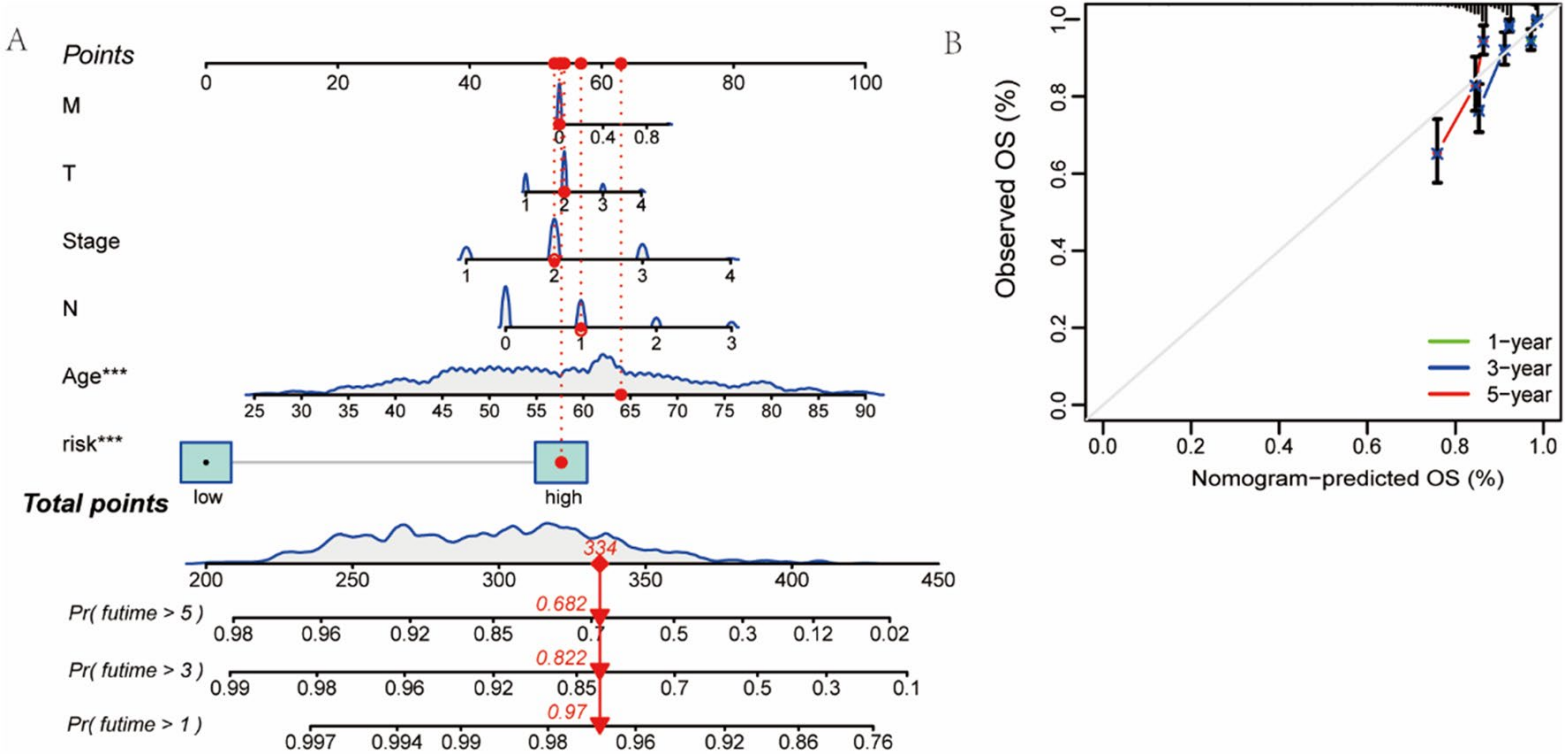
**A** Nomogram of patient. **B** Calibration curve analysis of the 1-, 3-, and 5-year survival prediction accuracy of the nomogram

Our PCA results showed that the model-related lncRNAs were more effective than the cuproptosis-related lncRNAs, cuproptosis-related genes, and all genes in distinguishing between different groups (Fig. 6A–D). Figure 7A, B displays the results of nomogram, which showed a good prediction of OS of patients with BC at 1, 3, and 5-year.

### Tumor mutation burden and survival analyses

**Fig. 8:**
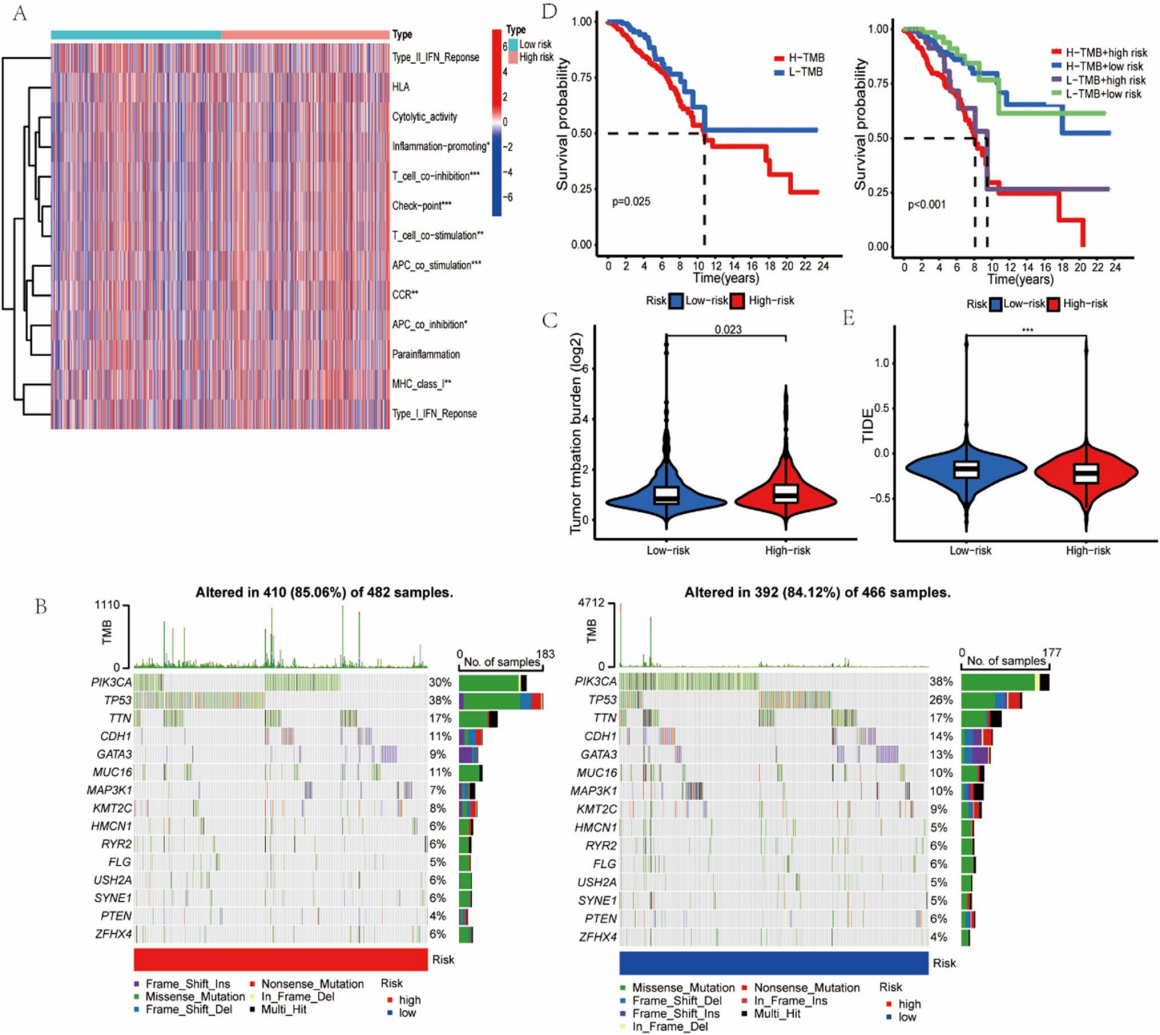
**A**, **B**, **C** and **E** respectively demonstrated the result of differences in immune function,tumor mutations, TMB and TIDE. **D** OS analysis of TMB combined with risk score

The results of tumor mutation are shown in Fig. 8B. According to the results, we discovered that patients with a higher TMB had a shorter OS, and that the higher TMB with the higher risk score had the shortest OS (Fig. 8C, D).

### Difference in immune function

The results of immune function demonstrated that inflammation-promoting, T cell co-inhibition, checkpoint, APC co-stimulation, T cell co-stimulation, CCR, APC co-inhibition and MHC class I responses were significantly increased in the high-risk group, which may increase the risk of death in patients with BC (Fig. 8A). Our analysis of TIDE showed that patients with higher risk scores performed better to immunotherapy than patients with lower risk scores (Fig. 8E).

### Relationship between risk score and drug sensitivity

**Fig. 9:**
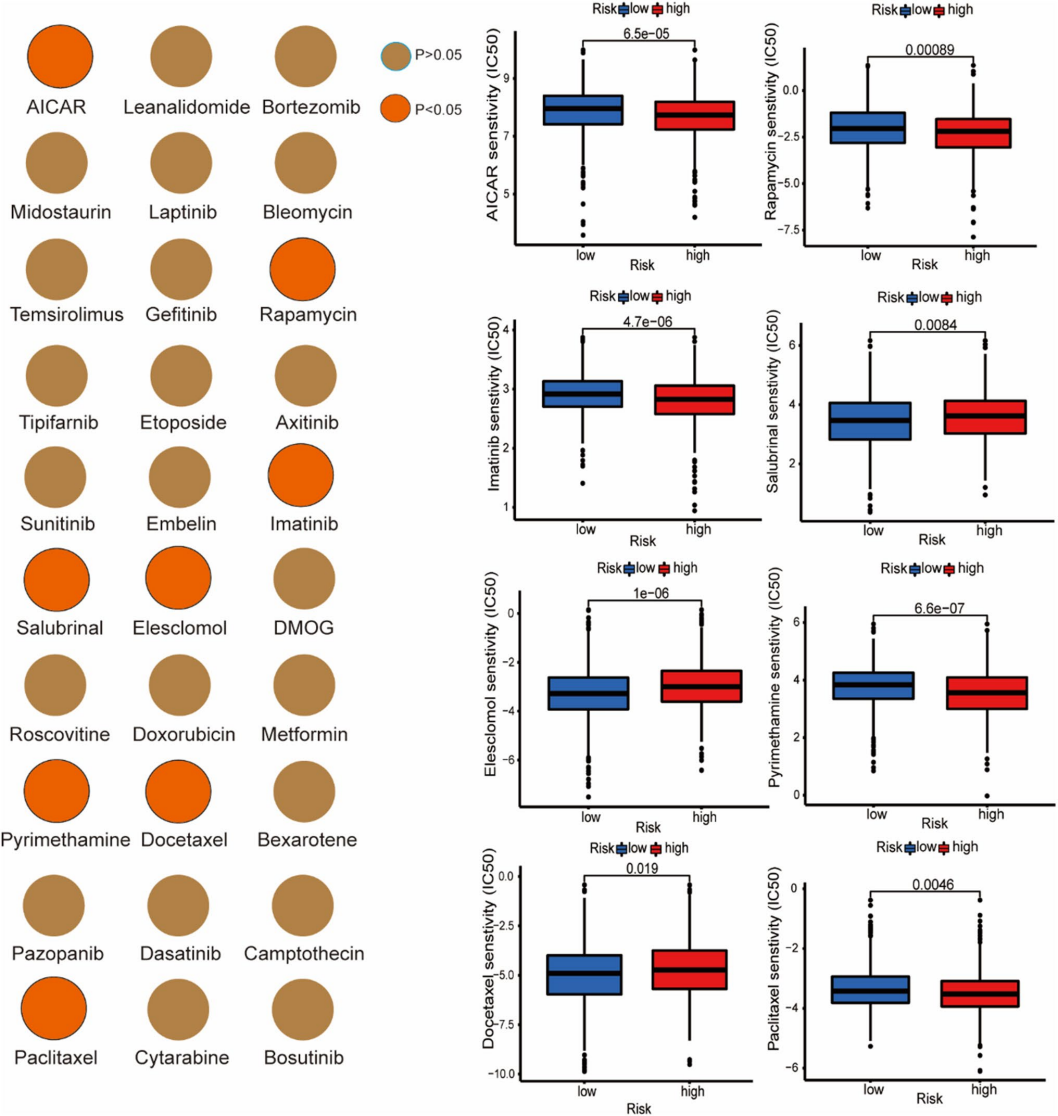
Relationship between risk score and drug sensitivity

Through the analysis of the sensitivity of 30 common antitumor drugs with different risk scores, we found that AICAR, Rapamycin, Imatinib, Pyrimethamine and Paclitaxel had lower IC50 in the high-risk group, indicating that the sensitivity of these drugs increased with the increase of risk scores (Fig. 9). On the contrary, Salubrial, Elescomol and Docetaxel had lower IC50 in the low-risk group, indicating that the sensitivity of these drugs decreased with the increase of risk scores (Fig. 9).

## Discussion

BC is among the top causes of death for women, and the incidence continues to rise, suggesting that we desperately need to improve our understanding of BC [24, 25]. As clearly demonstrated, clinicopathological features have proven capable of accurately assessing the cancer patient outcomes [26]. Therefore, we need to find other methods to better judge and identify the prognosis of patients. Numerous studies have shown that biomarkers screened by bioinformatics technology can predict the development and prognosis of cancer, which indicates that we can use this technology to deepen our understanding of BC [27–29].

Cuproptosis is a unique type of cell death due to copper triggering the aggregation of mitochondrial lipoacylated proteins and the instability of Fe-S cluster proteins. Studies have proved that cuproptosis-related genes can be used as a prognostic factor for renal cell carcinoma [30], while studies based on the correlation of cuproptosis in BC have not yet been conducted. There are many biological processes that are regulated by lncRNAs, including cell differentiation, apoptosis, and gene regulation [31], and studies have proved that lncRNAs control the progression, recurrence, and prognosis of cancer [32, 33]. Here, cuproptosis-related lncRNAs were identified using bioinformatics analysis, and nine key lncRNAs were obtained for prognostic model construction. Among the nine key cuproptosis-related lncRNAs we obtained, MME-AS1 has been confirmed to be related to the survival in intrahepatic cholangiocarcinoma [34], while OTUD6B-AS1 is not only associated the progression of various cancers, but also with tumor drugs resistance [35–37]. The remaining key lncRNAs have not been reported or investigated in literature. However, this study confirmed their role in the prognosis of BC, while other aspects of their function remain unstudied.

According to the risk formula obtained in this study, the higher the risk score of patients, the higher the mortality rate, and there were significant differences in PFS between different risk groups. Clinical practicability assessment of the model showed that it was capable of accurately predicting the prognosis of BC. Through calculating TMB, we found that patients with high TMB had a shorter OS than those with low TMB, while high-risk patients with high TMB had the shortest OS. We also compared the immune functions of the high and low risk groups, and found that the functions of the checkpoint and MHC class I were more active in the high-risk group, which may explain the shorter OS of the high-risk group. Moreover, immune checkpoint inhibitors have proved to have anticancer effects on various cancers [38, 39], which supports the reliability of our model. It has been reported that MHC class I deficiency can promote immunotherapy resistance in BC [40, 41], and the increased MHC class I response in the high-risk group in this study may explain the lower TIDE in high-risk patients. We also found that AICAR, Rapamycin, Imatinib, Pyrimethamine, Paclitaxel, Salubrial, Elescomol and Docetaxel have different IC50 in different risk groups through the sensitivity analysis of common anti-tumor drugs, indicating that the sensitivity of these drugs is different in different risk groups, which may provide guidance for different patients in the selection of anti-tumor drugs in clinical practice.

In this study, nine cuproptosis-related lncRNAs were used as the entry point to construct a prognostic model for BC. The AUC values of our model at 1 year, 3 years and 10 years are 0.783, 0.728 and 0.795 respectively, and these values are substantially higher than those of other models [42, 43], indicating that our cuproptosis-related lncRNAs prognostic model may be a more effective approach to predicting prognosis. However, there are still some limitations to our findings. First, we only used TCGA database, and our model needs to be verified in other databases. Second, we only used bioinformatics analysis without combining clinical and basic experiments to verify our model. However, it is undeniable that our model displayed promising performance as tool for prognosis prediction.

### Supplementary Information

**Additional file 1: Figure S1.** (A) LASSO regression graphs. Each curve in the figure represents the change trajectory of each independent variable coefficient, and the upper abscissa is the number of non-zero coefficients in the model. (B) LASSO regression cross-validation results. The red dotted line is the cross-validation curve, the upper and lower standard deviation curves along the λ series are error lines, and the vertical dotted line represents two selected λ. **Table S1.** The functional analysis results of the target lncRNAs.

## Data Availability

All data can be obtained from public databases. All data were downloaded from The Cancer Genome Atlas (TCGA).
