# Supplementary material for: A novel prognostic model of breast cancer based on cuproptosis-related lncRNAs
Source: Discov Oncol. 2024 Feb 14;15:35. doi: 10.1007/s12672-024-00888-3 (PMC10866837; doi:10.1007/s12672-024-00888-3)

Additional Figure 1 (A) LASSO regression graphs. Each curve in the figure represents the change trajectory of each independent variable coefficient, and the upper abscissa is the number of non-zero coefficients in the model. (B) LASSO regression cross-validation results. The red dotted line is the cross-validation curve, the upper and lower standard deviation curves along the λ series are error lines, and the vertical dotted line represents two selected λ.

Additional Table 1 The functional analysis results of the target lncRNAs.

Additional Figure 1


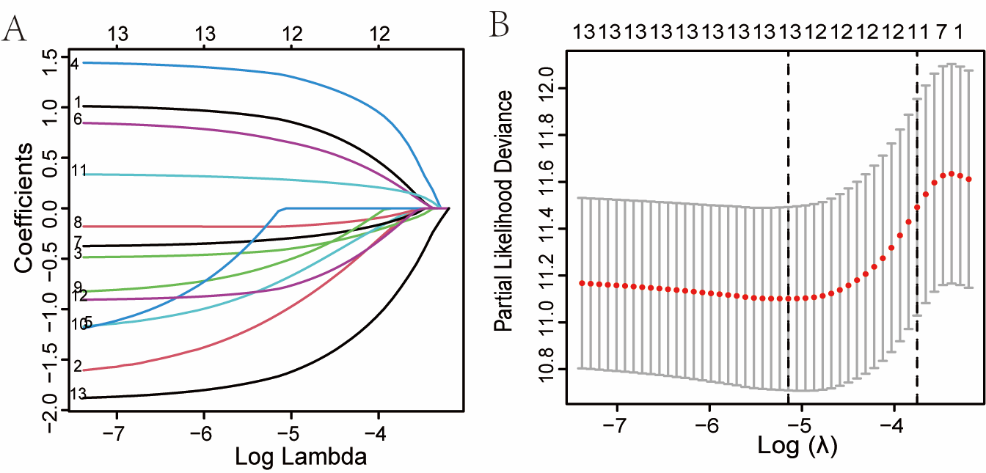


Additional table 1


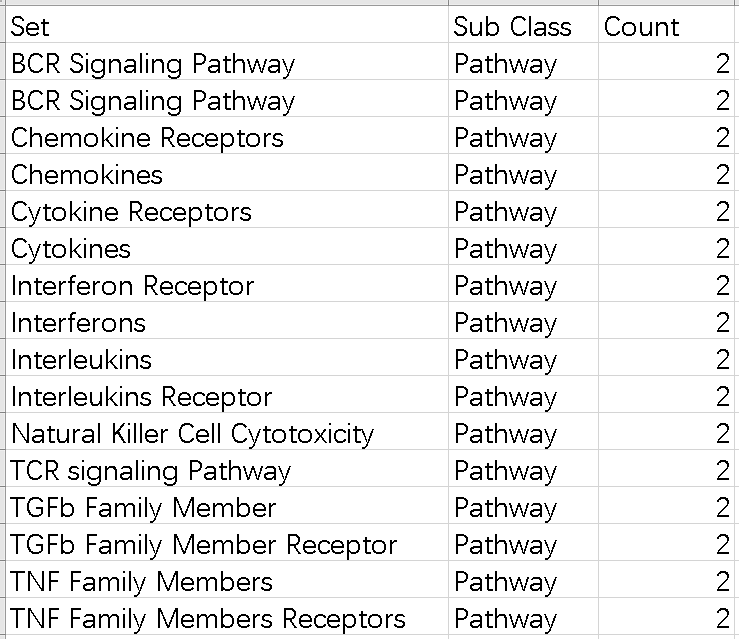

Supplement: Supplementary file 1 — Additional file 1: Figure S1. (A) LASSO regression graphs. Each curve in the figure represents the change trajectory of each independent variable coefficient, and the upper abscissa is the number of non-zero coefficients in the model. (B) LASSO regression cross-validation results. The red dotted line is the cross-validation curve, the upper and lower standard deviation curves along the λ series are error lines, and the vertical dotted line represents two selected λ. Table S1. The functional analysis results of the target lncRNAs. [file 12672_2024_888_MOESM1_ESM.docx]
